# Efficacy and Safety of Metronomic Chemotherapy Versus Palliative Hydroxyurea in Unfit Acute Myeloid Leukemia Patients: A Multicenter, Open-Label Randomized Controlled Trial

**DOI:** 10.31557/APJCP.2020.21.1.147

**Published:** 2020

**Authors:** Saranya Pongudom, Phichayut Phinyo, Yingyong Chinthammitr, Kanyaporn Charoenprasert, Harutaya Kasyanan, Klaijith Wongyai, Jittiporn Purattanamal, Naiyana Panoi, Anoree Surawong

**Affiliations:** 1 *Division of Hematology, Department of Internal Medicine, Udon Thani Hospital, Udon Thani, *; 2 *Center for Clinical Epidemiology and Clinical Statistics, Faculty of Medicine, Chiang Mai University, Chiang Mai, *; 3 *Division of Hematology, Department of Internal Medicine, Faculty of Medicine Siriraj Hospital, Bangkok, *; 4 *Division of Hematology, Department of Internal Medicine, Si Sa Ket Hospital, Si Sa Ket, *; 5 *Division of Hematology, Department of Internal Medicine, Buddhachinaraj, Hospital, Phitsanulok, *; 6 *Division of Hematology, Department of Internal Medicine, Sawanpracharak Hospital, Nakhon Sawan, *; 7 *Division of Hematology, Department of Internal Medicine, Maharaj Nakhon Si Thammarat Hospital, Nakhon Si Thammarat, *; 8 *Division of Hematology, Department of Internal Medicine, Chonburi Hospital, Chon Buri, *; 9 *Division of Hematology, Department of Internal Medicine, Sanprasithiprasong Hospital, Ubon Ratchathani, Thailand. *

**Keywords:** Acute myeloid leukemia, elderly, metronomic chemotherapy, hydroxyurea

## Abstract

**Background:**

Management of unfit AML patients is a therapeutic challenge. Most hematologists tend to avoid aggressive treatment leaving patients with a choice of best supportive care. We hypothesized that metronomic chemotherapy could be an alternative treatment for unfit AML patients.

**Methods:**

A multi-center randomized controlled trial was conducted in seven university-affiliated hospitals in Thailand. Unfit AML patients were recruited and followed up from December 2014 to December 2017. Patients were randomly assigned to receive either metronomic chemotherapy or palliative hydroxyurea. Overall survival rates were compared using Cox’s proportional hazard survival analysis.

**Results:**

A total of 81 eligible patients were randomly allocated and included for ITT analysis. The OS rate was higher in group receiving metronomic chemotherapy than in group receiving palliative treatment at 6 and 12 months with borderline significance (6 months HR 0.60; 95%CI 0.36, 1.02; p-value 0.060; 12 months: HR 0.66; 95%CI 0.41, 1.08; p-value 0.097).

**Conclusion:**

Metronomic chemotherapy could prolong survival time of unfit AML patients, especially in the first 12 months after diagnosis without increasing treatment-associated adverse events.

## Introduction

Acute myeloid leukemia (AML) is a heterogenous and fatal neoplastic disease which primarily affects more adults than children. The initial mainstay of therapy for younger patients is induction chemotherapy (Peyrade et al., 2012). For almost 50 years, the treatment outcome of unfit AML patients had only been slightly improved due to commonly known therapeutic challenges for this specific group of patients, treatment-resistance or intolerance and treatment-related mortality (Klepin and Balducci, 2009). Complete remission (CR) rates and disease-free survival time tended to decrease with increasing age (Appelbaum et al., 2006). Although the complete remission rate could be as high as 60%, the median overall survival for patients receiving intensive treatment was only 7-12 months and 2-year overall survival rate was 10-27% (Kantarjian et al., 2006; Gardin et al., 2007). Previous study reported that a quarter of elderly AML patients who received standard chemotherapy experienced early death from chemotherapy-related toxicity (Kantarjian et al., 2006).

Currently, there is no standard recommended treatment for unfit AML patients. The standard of care typically offered to unfit patients includes best supportive care, intensive chemotherapy and low-dose ara-C (Roboz, 2007). Generally, only 10-30% of older or unfit AML patients received standard chemotherapy regimen due to the aforementioned concerns. As the use of low-dose subcutaneous ara-C was neither practical nor widely used in Thailand, most patients were offered a choice of best supportive care or palliative treatment which showed a median survival time of only 2-3 months in other studies (Menzin et al., 2002; Oran and Weisdorf, 2012). Although several novel agents are being investigated such as Decitabine, Clofarabine, Gemtuzumab ozogamycin (GO), Farnesyl Transferase Inhibitors (FTIs) and FLT3 inhibitors, it has still not been endorsed for general use and cost-effective evaluation is warranted prior to becoming standard use in developing countries (Erba, 2007; Fenaux et al., 2009; Burnett, 2012). 

In the past decades, several clinical trials had established the potential efficacy of metronomic chemotherapy (MCT) in cancer patients. It consists of chronic administration of relatively low dose chemotherapeutic agents, preferably oral route, with least interruption or drug-free period (Pasquier et al., 2010). As metronomic chemotherapy had been shown to be potentially effective in both solid and hematologic malignancies including AML with significantly less toxicity compared to standard regimen (Kerbel and Kamen, 2004; Burnett et al., 2007; Kapoor et al., 2016).We hypothesized that metronomic chemotherapy would be superior to palliative care in improving overall survival of the patients without risking patients on more treatment-associated adverse events and complications. 

This multicenter, open-label, parallel-group, randomized controlled trial aimed to compare therapeutic efficacy and safety outcomes of metronomic chemotherapy compared to palliative treatment (using hydroxyurea) in unfit AML patients. 

## Materials and Methods

A multi-center, multi-regional, open-label, randomized controlled trial was conducted at 7 provincial tertiary care hospitals in Thailand (Udon Thani, Si Sa Ket, Phitsanulok, Nakhon Sawan, Chon Buri, Nakhon Si Thammarat and Ubon Ratchathani provincial hospitals) from December 2014 to December 2017. This study was financially supported by the Thai society of Hematology (TSH). The study protocol was approved by institutional review boards in each participating center. This trial was registered on Thai Clinical Trial Registry (TCTR 20150918001). Patient enrollment was done independently by the investigator in each site. Prior to trial participation, written informed consent was obtained from all eligible patients.

*Patients*

Eligible patients with histologically-confirmed diagnosis of acute myeloid leukemia (AML), according to WHO criterion, aged at least 55 years or older at time of diagnosis with Eastern Cooperative Oncology Group (ECOG) performance status of 0 to 4 who refused or were considered by attending hematologist as unfit to be treated with standard intravenous chemotherapy were enrolled. Patients who were unable to completely attend regular follow-up visits or had one of the following conditions: (1) congestive heart failure; (2) decompensated liver cirrhosis; (3) chronic kidney disease stage 3 to 4; (4) moderate to severe stage of chronic obstructive pulmonary disease; and (5) acquired immunodeficiency syndrome (AIDS) were excluded. All included patients were newly diagnosed and had never received any chemotherapy treatment prior to study enrollment. Any patients with emergency conditions requiring immediate resuscitation such as hyperleukocytosis, metabolic derangement, or systemic infection would be stabilized prior to randomization. 

*Randomization and allocation of treatment*

The recruited patients were randomly assigned into one of two treatment groups (metronomic chemotherapy group vs. palliative group) in one-to-one allocation ratio based on computer-generated random sequence with varying block size of 2, 4 and 6. Treatment allocations were issued by a central research assistant who was independent of the recruitment process via telephone calls. After treatment assignment, patients and attending hematologists were aware of the treatment assigned to the patient.

Patients in metronomic chemotherapy group received a 50 mg per m^2^ of Etoposide for 5 days plus 60 mg per m^2^ of 6-Mercaptopurine (6MP) for 2 weeks and 40 mg per m2 of prednisolone for 2 weeks. The regimen was orally administered every 21 days for 4 cycles. The patients were followed up for evaluation of response at 2nd and 4th cycle. 

Patients assigned to palliative hydroxyurea group were given an appropriate dosage of oral hydroxyurea to maintain number of white blood cell counts to less than 10,000 cell/mm^3^. The patients were scheduled for follow-up visit every 1 to 2 weeks. The treatment response was evaluated at 2^nd^ and 4_th_ months after initiation of treatment.

Routine blood samples were collected at each scheduled visit for complete blood count and blood chemistries (liver function test and renal function test). All patients were closely monitored for adverse events during the study period. Patients who survived after the first course of treatment would be repeated with the same protocols until primary endpoint occurred. Routine follow-up was appointed for all patients every three weeks to assess for the need of blood transfusion. 

*Outcomes measure*

The primary efficacy outcome was overall survival (OS), defined as the time from treatment assignment until death from any cause. As unfit AML patients in Thailand had a short life span, the author planned to examine the overall survival between both groups at 6 and 12 months after patient randomization, respectively. All patients were followed until death or study closure. In-hospital deaths were retrieved from medical records, others would be obtained from national death registry. Patients who were alive at the end of the study period were considered as censors. Additional analysis was done on the differences in median survival time among groups. 

Secondary outcomes were the occurrence of adverse events according to the National Cancer Institute Common Toxicity Criteria version 2.0 (i.e. nausea/emesis, diarrhea, mucositis, and clinical bleeding), incidence of febrile neutropenia, systemic infection, number of transfusion requirement, number of admissions and cumulative duration of hospitalization.

*Study size estimation*

The sample size was estimated based on a two-sample comparison of survival functions via Log-rank test. As most studies concerning metronomic chemotherapy in AML were single group, no hazard ratio was reported. Based on previous studies (Sandes et al., 2011), the hazard ratio of metronomic chemotherapy was estimated at 0.43. A sample size of 33 patients per group was powered enough to detect statistical significance with a two-sided 5% alpha error, a statistical power of 80%, and anticipated 25% dropout rate in each arm.

*Statistical analysis*

Descriptive statistics for normally-distributed continuous data were reported with mean and standard deviation. Median and interquartile range were used for skewed data. Frequency and percentage were used for summarizing categorical data. Continuous variables were compared using independent t-test. Fisher’s exact probability test was used for categorical variables. All test were two-sided and considered to be statistically significant when p-values were less than 0.05. 

The primary analysis was done in an intention-to-treat manner. The overall survival at 6 and 12 months were compared using both non-parametric Kaplan-Meier method and Cox proportional hazards regression. Kaplan-Meier curves were visualized, comparison of differences was done by log-rank test. The proportional hazard assumption was assessed via statistical testing using Schoenfeld residuals. To quantify treatment effect, hazard ratios (HRs) were estimated from the Cox’s regression model and were reported with 95% confidence intervals and p-values. The median survival times between treatment groups were estimated and compared using Laplace regression. 

The secondary safety outcomes were analyzed based on number of adverse events occurring over total follow-up time in each group (incidence rate with a unit of person-time). To estimate the size of effect, incidence rate differences were reported with 95% confidence intervals and p-values. No interim analysis for efficacy or safety was planned. All statistical analyses were performed with Stata statistical software version 15. 

## Results

*Patient characteristics*

**Figure 1:**
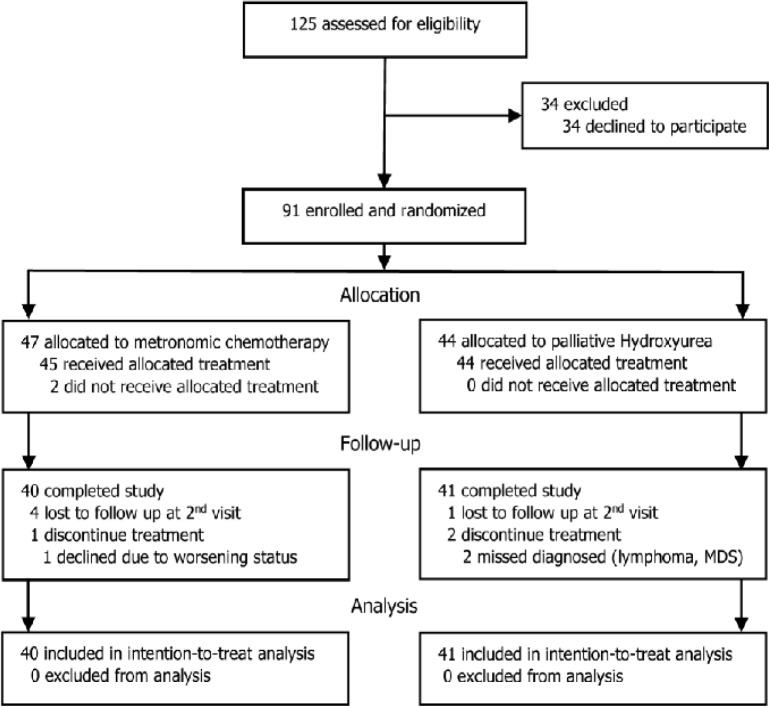
CONSORT Study Flow

A total of 91 eligible patients were randomly allocated into two treatment groups, 47 patients to metronomic chemotherapy group and 44 patients to palliative hydroxyurea group. Two patients did not receive the assigned treatment, one requested for intensive chemotherapy and another asked for best supportive care, their follow-up plan were discontinued. There were five patients who were lost to follow up after the first scheduled visit. One patient in metronomic chemotherapy group decided to leave the study after being admitted to intensive care unit due to severe pneumonia. Two patients in palliative hydroxyurea group were subsequently found to be misdiagnosed and had to leave the trial to receive their specific treatments. The remaining 81 patients with complete follow-up data, 40 in metronomic chemotherapy and 41 in palliative hydroxyurea, were included for ITT analysis (Figure 1). 

There were no significant differences in the patient baseline characteristics between two treatment groups (Table 1). The median age was 66 years old with 2:3 male to female ratio. Most of the patients included were ECOG 1 and 2 and only 13.6% had stable medical comorbidities. As some participants who initially presented with hyperleukocytosis were given hydroxyurea to lower the number of white blood cell counts before being randomized into one of two groups, the initial white blood cell counts were not different.

*Primary efficacy endpoints*

The study median follow-up time was 2.4 months with interquartile range of 1.2 and 4.7 months. The overall survival rate at 6 months was higher in metronomic chemotherapy group compared with palliative hydroxyurea group (HR 0.60; 95%CI 0.36, 1.02; p-value 0.060). At 12 months, the overall survival rate remained higher in metronomic chemotherapy group, but with less difference than that at 6 months (HR 0.66; 95%CI 0.41, 1.08; p-value 0.097). Both of the results showed borderline statistical significance.

**Figure 2:**
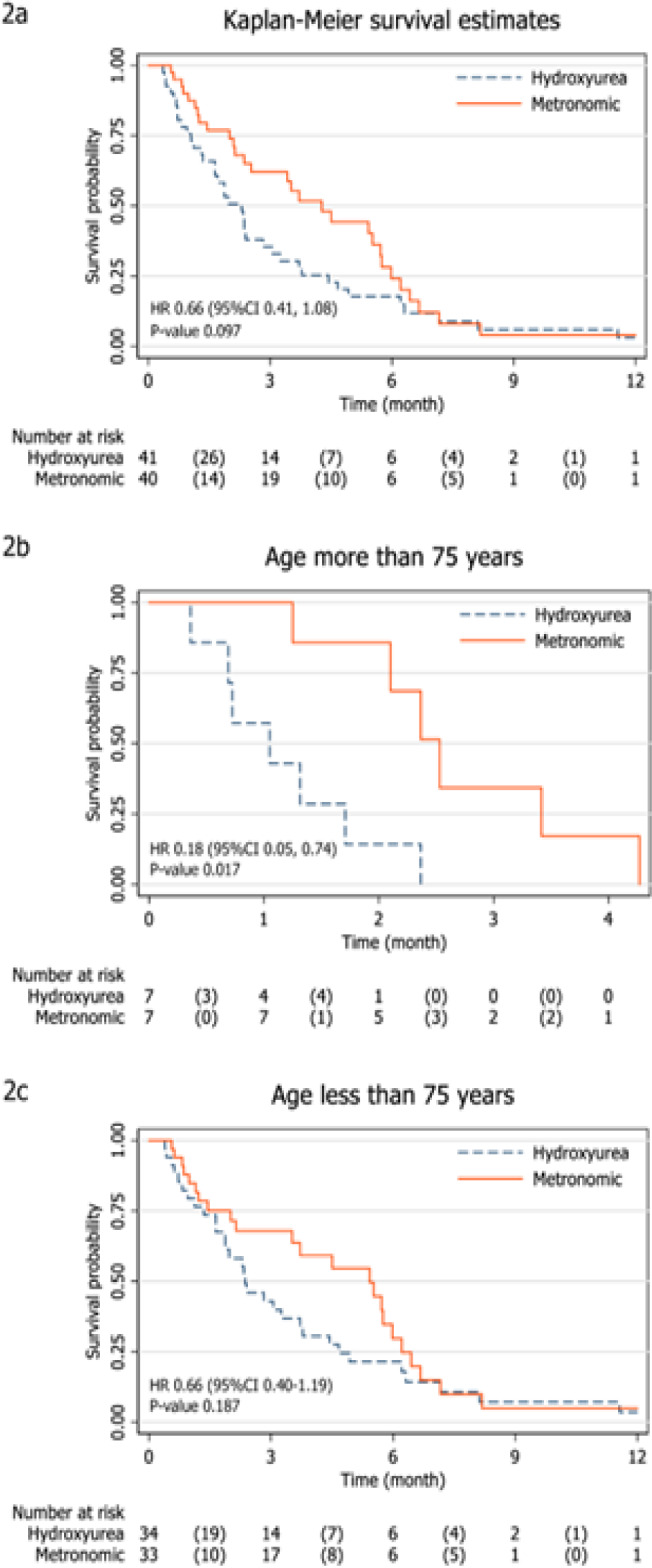
Kaplan-Meier Survival Estimates of Overall Survival in Unfit AML Patients Receiving Metronomic Chemotherapy (Solid Line) or Palliative Hydroxyurea (Dot Line) (2a). Comparison of Kaplan-Meier survival estimates of overall survival in unfit AML patients receiving metronomic chemotherapy or palliative hydroxyurea between patients aged more than 75 years old (2b) and less than 75 years old (2c).

The median survival time was substantially longer in group that received metronomic chemotherapy (4.03 vs 2.30 months) with a median survival time difference of 1.73 months (Median differences 1.73, 95%CI 0.38, 3.08, p-value 0.012) (Table 2, Figure 2).

*Secondary endpoints*

There were 324 adverse events in metronomic chemotherapy group and 228 events in palliative hydroxyurea group. The follow-up time was also longer in metronomic chemotherapy group compared to hydroxyurea group. Patients treated with metronomic chemotherapy tended to have higher incidence rate of febrile neutropenia than patients treated with hydroxyurea; however, the difference was not significant (0.85 vs 0.62, IRD 0.23, 95%CI -0.14, 0.60, p-value 0.116). In contrast, systemic infection tended to occur more frequently, although not significant, in group treated with palliative hydroxyurea than in metronomic chemotherapy group (0.78 vs 0.99, IRD -0.21, 95%CI -0.62, 0.20, p-value 0.156). 

Both treatment groups showed no significant differences in the occurrence of all National Cancer Institute-defined adverse events. Most common adverse events were grade 1 or 2 bleeding and mucositis. Grade 3 or 4 events rarely occurred in this study. Metronomic chemotherapy group had significantly a higher rate of transfusion requirements than palliative hydroxyurea group (2.24 vs 1.58, IRD 0.66, 95%CI 0.06, 1.26, p-value 0.002). Nonetheless, the number of admissions and cumulative duration of hospital stay in metronomic chemotherapy group did not differ significantly from those in hydroxyurea group (Table 3). 

**Figure 3:**
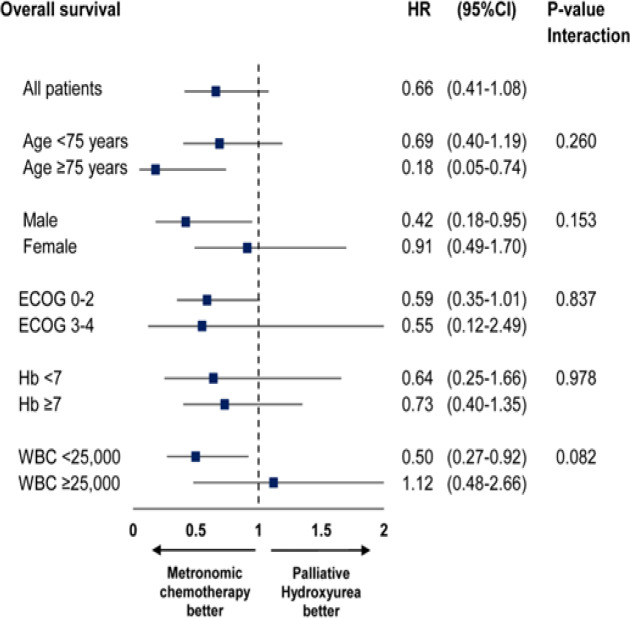
Subgroup Analysis Plot

Post hoc subgroup analyses for primary efficacy endpoint at 12 months revealed significant differential treatment effects according to patient’s age group, gender and white blood cell count prior to randomization (Figure 3). AML patients aged more than 75 years old, male gender and initial white blood cell count less than 25,000 cells/mm^3^ seemed to gain more benefit from metronomic chemotherapy. However, the test of interaction was insignificant. Kaplan-Meier survival curves of patients in two different age groups were shown (Figure 2b, 2c).

**Table 1:** Demographic and Cinical Characteristics of Participants by Treatment Group

	Metronomic chemotherapy (n=40)		Palliative Hydroxyurea (n=41)	
	n	(%)	n	(%)
Age (year, mean±SD)	66.4	**±**7.1	68.5	**±**7.7
Gender				
Male	17	42.5	17	41.5
Female	23	57.5	24	58.5
ECOG performance status				
ECOG 0	3	7.5	2	4.9
ECOG 1	21	52.5	20	48.8
ECOG 2	10	25.0	15	36.5
ECOG 3	5	12.5	4	9.8
ECOG 4	1	2.5	0	0.0
Comorbidities	6	15.0	5	12.2
Initial laboratory values				
Complete blood count				
Haemoglobin (g/dl, mean±SD)	8.1	**±**1.9	7.3	**±**2.7
WBC count (cells/mm^3^, mean±SD)	30,716.60	**±**66,083.90	35,045.90	**±**39,645.90
Platelet count (cells/mm^3^, mean±SD)	80,512.80	**±**84,615.20	93,315.00	**±**59,061.00
Blood chemistry				
Serum creatinine (mg/dl, mean±SD)	0.83	**±**0.28	1.19	**±**1.2
Total bilirubin (U/L, mean±SD)	0.86	**±**0.58	1.17	**±**1.12
AST (U/L, mean±SD)	32.8	**±**19.3	43.7	**±**36.5
ALT (U/L, mean±SD)	31.3	**±**25.6	37.1	**±**35.6

**Table 2:** Primary Efficacy Endpoints

	Metronomic chemotherapy (n=40)	Palliative Hydroxyurea (n=41)	Hazard ratio	95% Confidence interval	P-value
6-month OS rate	24.2	17.7	0.60	(0.36,1.02)	0.060
(% , 95%CI)	(10.3,41.1)	(7.8, 30.9)			
12-month OS rate	4.03	2.95	0.66	(0.41,1.08)	0.097
(% , 95%CI)	(0.30,17.00)	(0.23,12.91)			
Median survival time	4.03	2.30	1.73†	(0.38,3.08)	0.012
(month, 95%CI)	(2.92,5.14)	(1.54,3.06)			

† Median survival time differences

**Table 3:** Adverse Events, Chemotherapy-Associated Toxicity and Hospitalization Requirements by Treatment Group

Event	Metronomic chemotherapy		Palliative Hydroxyurea		Incidence rate difference	95% Confidence interval	P-value
	Number of events (incidence rate)						
Total number of events	324		228				
Total Follow-up time, day	4,374		3,739				
Adverse events / Toxicity							
Febrile neutropenia	37	(0.85)	23	(0.62)	0.23	(-0.14,0.60)	0.116
Systemic Infection	34	(0.78)	37	(0.99)	0.21	(-0.62,0.20)	0.156
Transaminitis	6	(0.13)	5	(0.13)	0.01	(-0.16,0.16)	0.487
Acute kidney injury	7	(0.16)	5	(0.13)	0.02	(-0.14,0.19)	0.387
Diarrhea							
Grade 1-2	9	(0.21)	3	(0.08)	0.13	(-0.04,0.29)	0.077
Grade 3-4	1	(0.02)	1	(0.03)	0.01	(-0.07,0.07)	0.461
Bleeding							
Grade 1-2	10	(0.23)	14	(0.37)	0.15	(-0.39,0.10)	0.119
Grade 3-4	5	(0.11)	2	(0.05)	0.06	(-0.06,0.19)	0.194
Nausea/Emesis							
Grade 1-2	5	(0.11)	3	(0.08)	0.03	(-0.10,0.17)	0.327
Grade 3-4	1	(0.02)	0	0	0.02	(-0.02,0.07)	0.27
Mucositis							
Grade 1-2	13	(0.3)	6	(0.16)	0.14	(-0.07,0.34)	0.107
Grade 3-4	1	(0.02)	2	(0.05)	0.03	(-0.12,0.06)	0.27
Transfusion requirements	98	(2.24)	59	(1.58)	0.66	(0.06,1.26)	0.002
Hospitalization requirements							
Admission	97	(2.22)	68	(1.82)	0.4	(-0.22,1.02)	0.105
Cumulative duration of hospital stay (day, mean±SD)	7.68	13.95	**±**6.41	12.29	**±**1.26	(-4.55,7.07)	0.667

## Discussion

Optimal management for unfit acute myeloid leukemia patients remains an important clinical dilemma (Peyrade et al., 2012). Several clinical factors other than age, performance status, comorbidities and cytogenetics such as bilirubin, neutrophil count, albumin, hemoglobin and creatinine were reported to be associated with treatment-related mortality (Estey et al., 1989; Appelbaum et al., 2006). Many multivariable scoring systems were developed to aid physician in prediction of treatment-related mortality in each patient (Walter et al., 2011). As some specific subgroups of older patients could still benefit from intensive chemotherapy, estimating medical fitness for treatment tolerance based on these assessment tools should be a basis of evaluation prior to the treatment assignment (Walter and Estey, 2015). However, this concept was not widely applied in Thailand, and many resource-limiting countries. 

Generally, AML patients who were unfit for standard induction therapy were offered best supportive care (consisting of hydroxyurea, antibiotics and transfusions) and low-intensity chemotherapy regimen such as cytarabine 100 mg/m^2^ for 5 days plus idarubicin 10 mg/m^2^ for 2 days or subcutaneous low-dose ara-C in some institutions (Roboz et al., 2012). In the past decade, metronomic chemotherapy showed up as a potential treatment for various types of refractory malignancies including leukemia especially in developing countries (Pasquier et al., 2010; André et al., 2013). The proposed target of metronomic chemotherapy includes three main components of tumor microenviroment which are the cancer cells, the tumor vasculature and the immune system, some considered the strategy as multi-targeted therapy (Pasquier et al., 2010). However, the main mechanism of the regimen in treating hematologic malignancies including AML is assumed to be the inhibition of tumor angiogenesis (Padró et al., 2000). Few observational studies and one case report had revealed favorable survival outcome of using metronomic chemotherapy in AML patients (Banavali et al., 2005; Tandon et al., 2013; Kapoor et al., 2016). To our knowledge, this study is the first randomized controlled trial to directly examine the efficacy and safety outcomes of metronomic chemotherapy in unfit AML patients. 

In our study, the median age of the patient was 66 years whereas half of the patient had an ECOG performance status of 2. The median survival time in palliative hydroxyurea group was 2 months versus 4 months in metronomic chemotherapy group. This finding was in concordance to a retrospective cohort based on Surveillance, Epidemiology and End Results program (SEER) database which reported the median overall survival of untreated AML patient at 2 months and 6 months for treated AML patients (Oran and Weisdorf, 2012). Although the survival time in the untreated or palliative group were roughly the same, the survival time in treated group from SEER analysis seems to be better. This difference was due to the fact that cancer-patient survival is prone to be lower in developing countries due to inferior health care infrastructure and socioeconomic restrictions which finally leads to delayed diagnosis, late treatment initiation, limited access to effective treatment, and poor quality of supportive care (e.g. limited number of isolation units) (Jemal et al., 2011; André et al., 2013). Another reason was the result from SEER was not based on low-intensity treatment strategies or metronomic chemotherapy. A single group pilot study in AML patients with the same metronomic chemotherapy regimen (prednisolone, etoposide and 6-mercaptopurine) observed high response rate and a higher median survival time of 7-8 months which was probably due to higher proportions of younger participants (mean age 34.8 years; range 6-70 years) (Singh et al., 2017). A prospective study in India reported 6 months overall survival in patients treated with oral 6-mercaptopurine 75 mg/m^2^ per day (Kapoor et al., 2016). Although the survival seems to be longer than our study, the study was uncontrolled, non-randomized study with different metronomic regimen. 

From Cox’s regression, metronomic chemotherapy provided better 6 and 12 month overall survival than palliative therapy with borderline statistical significance. However, this cannot be concluded based on only statistical aspect as the power of study was possibly limited but also in term of clinical significance. As mentioned before, the median survival in unfit AML patients is estimated at 2-3 months in developing countries. Based on our study, metronomic chemotherapy doubled the median survival time of patients who received only best supportive care and potentially improved 6-month overall survival rate by 40 percent.

Intensive chemotherapy treatment could prolong survival in some subgroups of patients but with higher probability of treatment-associated adverse outcomes such as febrile neutropenia and severe systemic infection resulting in frequent and longer hospitalization. One study from the United States reported rates of expected serious treatment-associated events in AML patients on standard chemotherapeutic regimen that all patients developed grade 3 to 4 cytopenias during follow up and almost all, 90 percent, had a febrile neutropenic episode (Atallah et al., 2007). In the current trial, febrile neutropenia and systemic infection occurred 40 percent equally in each groups with an incidence rate lower than 1 per day of follow up. Acute kidney injury and transaminitis occurred in 10 percent of patients in both groups which was significantly less than previously mentioned figures in intensive chemotherapy study. 

Other documented adverse events also showed non-significant difference between groups except for transfusion requirements which was significantly increased in metronomic group. This might be attributed from the effect of myelosuppression from chemotherapeutic agents within the regimen (Singh et al., 2017). However, the total duration of hospital stay did not differ between groups. In a clinical setting where transfusion could be done in outpatient care, this could also lead to decrease in numbers of admission. This evidence suggested that metronomic chemotherapy was probably less toxic for unfit AML patients than standard induction chemotherapy and was comparably safe as palliative treatment (without chemotherapeutic agents). 

Subgroup analysis for primary efficacy end point at 12 months was done based on specific age cut-off point of 75 years due to shorter life expectancy, more frail to treatment related side effects , higher proportion of unfavorable cytogenetics and multidrug resistance (Appelbaum et al., 2006; Ossenkoppele and Löwenberg, 2015). Post hoc subgroup analysis revealed that, in our study, patients aged more than 75 years yielded better overall survival outcome than patients younger than 75 years. It is possible that metronomic chemotherapy shared the same features of attenuated chemotherapy regimen which allowed vulnerable groups of patients to prevail negative impacts of intensive treatments and gain partial benefit from minimally optimum doses of chemotherapy (Huang et al., 2014). Nonetheless, our study was not powered enough to test and conclude this interaction effect. 

In terms of gender, subgroup analysis suggested more beneficial effect from metronomic chemotherapy in male patient than in female. Female gender had been reported as potential risk factor of poor treatment outcomes and possible indicator of unfavorable cytogenetics in AML, although the mechanism was still unclear (Marcucci et al., 2005; Singh et al., 2017). Female hormonal variations could play a potential role in altering biology of the disease (Acharya et al., 2018). Previous studies had reported white blood cell counts at diagnosis as an independent risk factor for relapse and poor CR achievements in AML patients with specific cytogenetics (Martín et al., 2000; Cairoli et al., 2013). However, the cut-off point for marked hyperleukocytosis was commonly defined at >100,000 cell/mm^3^. In the present study, the data of white blood cell count at diagnosis was not available as all patients with hyperleukocytosis at presentation would be treated prior to trial recruitment and randomization, thus numbers of white blood cell prior to treatment allocation were used instead. One study reported white blood cell count >30,000 cell/mm^3^ as independent factor that had significant impact on overall survival of AML patients (Djunic et al., 2012). It was observed that patients with lower initial white blood cell counts tended to have a stable level of white blood cell counts during follow-up and had better overall prognosis than patients with higher initial white blood cell counts. 

The randomized design is the major advantage of our study and as mentioned above this study is the first done in this particular subject. Treatment allocation was centrally given and varying block technique was used in sequence generation. The study framework and protocol was pragmatic or routine-based. Multi-center recruitments also improves generalizability of study results. As there was no standard definition of unfit patients, we relied the eligibility of our patients on operational definition by attending hematologists on each study sites. The initial investigation was done as routine without any addition extras e.g. chromosomal study which was not done in every patients and was not considered in this trial. But this could also be justified as limitation of the trial in patient risk assessment.

There were some other limitations to be considered. Some baseline data were not recorded such as cytogenetic result, blast count and comorbities index as it was not routinely done in this domain of patients. Post-randomization exclusion was present due to misdiagnosed and patient-intended withdrawal. However, there was no crossover between groups and intention-to-treat was still fulfilled. We did not follow the patient for complete remission (CR) as unfit AML patients tended to have short survival time and the main objective of the treatment was to prolong survival, thus CR rate was not of particular interest as treatment that increases CR rates does not necessarily prolong survival time (Burnett et al., 2013). Another important aspect that this study did not measure was quality of life (QOL). Any further research should plan to evaluate this aspect of the patients. Lastly, metronomic chemotherapy is an emerging concept of cancer treatment with a wide range of treatment regimens. The formula used in this study could be adapted or changed, it is possible that unfit AML patients could benefit even more from other metronomic regimen. 

In conclusion, metronomic chemotherapy prolongs overall survival in unfit AML patients compared to palliative treatment with hydroxyurea with borderline significance. Metronomic chemotherapy should be considered as another option for treatment in AML patients who were considered unfit for standard induction chemotherapy, especially in resource-limiting countries, due to its favorable efficacy outcome, acceptable safety profile, and economical issue. 
